# Genomic Insights Into Carnivorous Fish in the Characiformes

**DOI:** 10.1002/ece3.73478

**Published:** 2026-04-13

**Authors:** Shihu Zhao, Xuesong Mei, Zhihao Zhang, Tian Xia, Shengyang Zhou, Yuehuan Dong, Shangbin He, Zhicheng Yao, Yuchun Li, Xiufeng Yang, Honghai Zhang

**Affiliations:** ^1^ School of Life Sciences Qufu Normal University Qufu Shandong China; ^2^ Shandong Key Laboratory of Wetland Ecology and Biodiversity Conservation in the Lower Yellow River Qufu Normal University Qufu Shandong China; ^3^ Zibo Municipal Bureau of Natural Resources and Planning Zibo Shandong China; ^4^ Yantai Forest Resources Monitoring and Protection Service Center Yantai Shandong China

## Abstract

The Characiformes exhibit remarkable dietary diversity, with carnivorous species displaying unique adaptations to high‐protein and high‐fat diets. However, the molecular mechanisms underlying this carnivorous specialization remain poorly understood. In this study, we adopted comparative genomic approaches using 12 high‐quality Characiformes genomes (covering nine subfamilies) with zebrafish as an outgroup. We first re‐annotated the genomes of five Characiformes species, subsequently performing phylogenetic reconstruction, gene family expansion and contraction analysis, candidate gene identification, and amino acid site substitution analyses. Enrichment analysis revealed that expanded gene families, positively selected genes, and rapidly evolving genes in Characiformes were predominantly linked to amino acid metabolism, lipid absorption and transport, energy metabolism regulation, and chitin‐related. Furthermore, amino acid substitutions in *phospho1* and *hsd17b7* were found to lead to substantial alterations in protein 3D structures, potentially modifying their biological functions associated with lipid metabolic homeostasis. These candidate genes are hypothesized to facilitate the efficient absorption and utilization of high‐protein and high‐fat diets by carnivorous Characiformes while mitigating adverse effects. Our findings reveal the molecular basis of carnivorous adaptation in Characiformes, providing a foundation for future functional validation and novel insights into dietary diversification in teleost fish.

## Graphical abstract

This study employed comparative genomic approaches using 12 high‐quality Characiformes genomes (covering nine subfamilies) with zebrafish as an outgroup to explore the molecular mechanisms underlying the carnivorous adaptation of Characiformes. Expanded gene families, positively selected genes, and rapidly evolving genes in carnivorous species were predominantly enriched in pathways related to amino acid metabolism, lipid absorption and transport, and energy metabolism regulation, while amino acid substitutions in phospho1 and hsd17b7 altered protein 3D structures and potentially modulated lipid metabolic homeostasis. These findings uncover the molecular basis for carnivorous Characiformes to efficiently utilize high‐protein and high‐fat diets, providing novel insights into dietary diversification in teleost fish and a foundation for future functional validation.

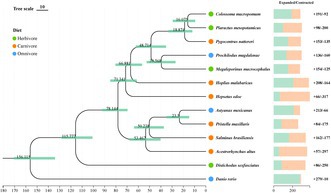

## 1 Introduction

The order Characiformes comprises over 2300 species, ranking among the most diverse groups of vertebrates (Malabarba and Malabarba 2020). Taxonomically, previous studies have classified Characiformes into two suborders: Characoidei and Citharinoidei (Nelson et al. 2016). Morphologically, this group is characterized by having adipose fins and well‐developed scales. Additionally, most species possess well‐developed multi‐cuspid jaw teeth, these may be attached along the margins of the oral jawbones or even extend beyond the oral cavity (Yang et al. 2023). At least in their adult stages, they lack barbels. Collectively, these traits constitute the primary diagnostic characteristics for identifying Characiformes. Body size exhibits considerable interspecific variation: the majority of species reach approximately three cm in length, while the largest representatives of the group can exceed one meter (Weitzman and Vari 1988). Equally diverse are body shapes, ranging from slender and streamlined to highly laterally compressed. Geographically, the vast majority of Characiformes are endemic to tropical freshwater regions in South America, with a small subset of species distributed in Africa (Malabarba and Malabarba 2020). These fishes occupy a broad range of ecological niches in their respective ecosystems and play an important role in maintaining community structure and stabilizing ecosystems (Taylor et al. 2006).

Similarly, the feeding habits of Characiformes exhibit remarkable trophic diversity, encompassing nearly all dietary types found in freshwater fish groups. Some are specialized piscivores, preying on small fish, while certain aggressive carnivores (e.g., *Salminus brasiliensis* ) even target small vertebrates like frogs, rodents, and birds (Graciano et al. 2022; NCN RO 2009). Other species are herbivores, feeding on aquatic plants, algae, and fruits and seeds that fall into the water column. These herbivores serve as important seed dispersers in their respective ecosystems (Calcagnotto and DeSalle 2009). A subset of benthic‐dwelling species are omnivores, feeding on algae, organic detritus, and invertebrates (e.g., beetles, worms). Notably, some members of the Citharinoidei exhibit a unique and highly specialized feeding behavior termed pterygophagy, in which they bite off and consume small fragments of fin tissue from other fish species (Lavoué et al. 2017). Several studies have indicated a close correlation between the feeding habits of fish and their morphological characteristics (Cooper and Westneat 2009). For example, most carnivorous fish possess conical teeth, while gape size, jaw position, and jaw orientation typically correlate with the size of their prey (Wainwright and Richard 1995; Winemiller et al. 2015). These carnivorous fish exhibit excellent burst swimming performance, which facilitates ambushing prey (Webb 1984). Additionally, many carnivorous ray‐finned fish have significantly shorter intestinal tracts and smaller abdominal cavities than their herbivorous counterparts (Burns 2021).

The dietary diversity of Characiformes serves as a key driver of their ecological success in diverse aquatic ecosystems, making them an ideal taxon for investigating the adaptive evolution of feeding strategies. An existing study utilizing mitochondrial and nuclear genes has shown that the ancestral diets within the Citharinoidei encompassed pelophagy/planktivory, omnivory, and invertivory. Specialized herbivory, carnivory, and pterygophagy may have evolved from this invertivorous ancestral diet (Lavoué et al. 2017). With the rapid development of genome sequencing technology, it has become possible to elucidate the molecular mechanisms underlying major phenotypic traits in animals. Recent research has generated high‐quality genomes for four Characiformes species (including the first genome of a Citharinoidei), revealing a paraphyletic evolutionary relationship within Characiformes. Additionally, this study identified copy number variations (CNVs) in genes (e.g., *scpp*) and specific gene deletions, providing key insights into the genetic basis underlying the unique dental morphology characteristic of Characiformes (Yang et al. 2023). Previous studies have identified a subset of genes and signaling pathways associated with dietary differences, including those in mammals and the four major Asian domestic carps (Kim et al. 2016; Wang et al. 2024). However, the molecular mechanisms underlying the diverse dietary traits of members within the diverse Characiformes, with a particular focus on those related to exclusive carnivory, remain insufficiently investigated.

In this study, we conducted comparative genomic analyses using 12 currently available high‐quality Characiformes genomes. First, we characterized expanded gene families, positively selected genes (PSGs), and rapidly evolving genes (REGs) among the exclusively carnivorous species in this dataset. Second, we identified molecular differences between specialized carnivorous and herbivorous taxa using an amino acid site substitution method. The findings of this study are expected to provide novel insights into the molecular mechanisms driving the evolution of dietary habits in Characiformes.

## 2 Methods

### 2.1 Genomic and Dietary Habits Data Collection

Genome sequences of Characiformes were retrieved from the NCBI database. However, most did not reach chromosome‐level quality. Across all these sequences, we assessed genome completeness using BUSCO (Benchmarking Universal Single‐Copy Orthologs, v5.2.2 (Simão et al. 2015)) with the “actinopterygii_odb10” database and default parameters (Table S1). Ultimately, we selected 12 high‐quality and relatively complete Characiformes genomes with BUSCO scores of the genome assemblies mostly above 90%, together with one outgroup species (zebrafish, *Danio rerio* , GenBank accession number: GCA_049306965.1) for our analysis. To verify the dietary habits of Characiformes, we collated data from the following databases: FishBase (Froese, R. & D. Pauly, Eds. 2025), Fishipedia (https://www.fishi‐pedia.com), Seriouslyfish (https://www.seriouslyfish.com), as well as several prior studies (Burns 2021; Araujo et al. 2021; Martinez et al. 2022; Berdugo and Narváez Barandica 2014; Ferreira et al. 2014; Murray et al. 2025). Subsequently, to support subsequent analyses, we divided these Characiformes into three dietary groups: carnivorous, omnivorous, and herbivorous. Detailed dietary habit and genomic information for the 12 Characiformes species are presented in Table 1.

**TABLE 1:** List of genomic information for Characiformes.

Scientific name	Family	Accession number	Scaffold N50	Trophic classification	Busco (%)
*Acestrorhynchus altus*	Acestrorhynchidae	GCA_034509495.1	34.1 Mb	Carnivore	81.6
*Astyanax mexicanus*	Characidae	GCA_023375975.1	51.9 Mb	Omnivore	100.0
*Colossoma macropomum*	Serrasalmidae	GCA_904425465.1	40.2 Mb	Herbivore	99.8
*Distichodus sexfasciatus*	Distichodontidae	GCA_032718565.1	1.0 Mb	Herbivore	78.2
*Hepsetus odoe*	Hepsetidae	GCA_017165825.1	25.8 Mb	Carnivore	81.2
*Hoplias malabaricus*	Erythrinidae	GCA_029633855.2	53.1 Mb	Carnivore	97.6
*Megaleporinus macrocephalus*	Anostomidae	GCA_021613375.1	45.0 Mb	Herbivore	94.2
*Piaractus mesopotamicus*	Serrasalmidae	GCA_040208305.1	47.2 Mb	Herbivore	87.3
*Pristella maxillaris*	Characidae	GCA_045781885.1	42.8 Mb	Carnivore	96.5
*Prochilodus magdalenae*	Prochilodontidae	GCA_024036415.1	348.3 Kb	Omnivore	84.9
*Pygocentrus nattereri*	Serrasalmidae	GCA_015220715.1	42.3 Mb	Carnivore	99.8
*Salminus brasiliensis*	Bryconidae	GCA_030463535.1	40.8 Mb	Carnivore	98.6

### 2.2 Genome Annotation

Among the 12 selected genomes, five species ( *Acestrorhynchus altus* , *Distichodus sexfasciatus* , *Hepsetus odoe* , *Piaractus mesopotamicus* , *Prochilodus magdalenae* ) only had raw assembly sequences without corresponding annotation information. To address this limitation, we conducted genome annotation for these five species.

We constructed species‐specific *de novo* repeat libraries using RepeatModeler v2.0.1 (Flynn et al. 2020). Subsequently, RepeatMasker v4.1.0 (Chen 2004) was employed to identify known and novel transposable elements (TEs) by aligning genomic sequences to both the species‐specific de novo repeat libraries and the Repbase database. We predicted protein‐coding genes by combining two complementary strategies: de novo gene prediction and homology‐based prediction. First, gene structures were predicted utilizing internal gene models with Augustus v2.5.5 (Stanke and Waack 2003), GlimmerHMM v3.0.4 (Majoros et al. 2004), and Geneid v1.4.4 (Parra et al. 2000). Second, protein sequences from five species ( *Astyanax mexicanus* , *Colossoma macropomum* , *Danio rerio* , *Hoplias malabaricus* and *Pygocentrus nattereri* ) were retrieved as templates for homology‐based prediction and aligned to the assembled genomes using Miniprot v0.12 (Li 2023). Finally, non‐redundant gene sets were constructed using EVidenceModeler v2.1.0 (Haas et al. 2008) by integrating de novo and homology‐predicted genes. Short genes (< 50 amino acids) and prematurely terminated genes were filtered out from the non‐redundant gene sets to yield the final complete gene sets. For the final annotation results, we evaluated their quality using BUSCO v5.2.2 with the “actinopterygii_odb10” database and default parameters.

### 2.3 Orthologous Gene Identification, Phylogenetic Tree Construction, and Gene Family Identification

To infer the phylogenetic relationships within Characiformes, we generated genomic datasets and conducted phylogenetic analyses to explain their evolutionary history. OrthoFinder v2.4.0 (Emms and Kelly 2015), combined with the Diamond (Buchfink et al. 2015) algorithm, was employed to identify “one‐to‐one” orthologous genes across all included genomes. Amino acid sequences of single‐copy orthologs were aligned using MUSCLE v3.8.31 (Edgar 2004). The sequence alignments were then converted to codon alignments using the Perl script PAL2NAL (Suyama et al. 2006). Phylogenetic trees were constructed from the single‐copy ortholog alignments using RAxML v8.1.12 (Stamatakis 2014) with parameters: ‐m GTRGAMMA ‐f a ‐x 12,345 ‐N 1000 ‐p 12,345 ‐T 64, employing the GTR‐GAMMA substitution model. Node support was assessed with 1000 bootstrap replicates. The divergence time within Characiformes was estimated using the MCMCTREE program in PAML v4.7 (Yang 1997) with parameters: ‐clock 2 ‐alpha 0.5 ‐model 3. Time calibration points were used to calibrate the temporal scale during computation, and all were sourced from the TimeTree database (https://www.timetree.org) (Kumar et al. 2017) to ensure computational accuracy.

We analyzed gene family expansions and contractions using CAFE5 (Mendes et al. 2020). The gene family clustering file generated by OrthoFinder was used as the primary input dataset, and outlier gene families (defined as families containing ≥ 100 genes in any single species) were systematically removed. Concurrently, the phylogenetic tree generated by MCMCTREE was adopted as the secondary input file (Lyu et al. 2025). A random birth‐and‐death model was used to estimate the size of the gene families at the ancestral nodes. Explicit modeling of rate variation between families was carried out using gamma distribution rate category modeling. To obtain a better fit to the data, we used the GAMMA model, compared the Model Gamma Final Likelihood, and determined that *k* = 4 was a better fit to the data by selecting the highest likelihood converging model. The running parameters were set as follows: ‐i gene_families_filter.txt ‐t tree.txt ‐p ‐k 4 ‐c 30 ‐o out (Zhang et al. 2023). We summarized the counts of expanding and contracting gene families for each species. Gene families with a significant *p* < 0.05 at any node were classified as significantly expanded or contracted. We statistically identified shared orthogroups (OGs) among five carnivorous Characiformes species: *Acestrorhynchus altus* , *Hepsetus odoe*, *Hoplias malabaricus* , *Pygocentrus nattereri* , and *Salminus brasiliensis* . It is noteworthy that although *Pristella maxillaris* is categorized as carnivorous in FishBase, it has a body length of ~4 cm, and its diet is dominated by worms and zooplankton, with no fish included (Murray et al. 2025). Therefore, we selected five other ferocious carnivorous Characiformes species that prey on fish for subsequent analyses.

### 2.4 Selective Pressure Analysis

We utilized the codeml program in PAML v4.7 software package to analyze all obtained one‐to‐one orthologues, aiming to assess the impact of natural selection on genes linked to carnivory. We assessed positive selection by calculating the ratio (ω) of the nonsynonymous substitution rate (Ka) to the synonymous substitution rate (Ks): *ω* > 1 indicates positive selection, *ω* < 1 indicates purifying (negative) selection, and *ω* = 1 indicates neutral evolution. First, we designated the five aforementioned ferocious carnivorous Characiformes species as the foreground branch, with the remaining Characiformes species and zebrafish (outgroup) as the background branch. For this analysis, we employed the branch‐site model (Ma: model = 2, NSsites = 2, fix_omega = 0; null model Ma0: model = 2, NSsites = 2, fix_omega = 1, omega = 1) to identify positively selected genes (PSGs) in carnivorous Characiformes. We performed a comprehensive screen for each gene. To minimize false positive results, we excluded genes with positive selection signals at 3 or more consecutive sites, genes with positively selected sites at the start or end of the coding sequence, and genes with positively selected sites with a Bayes Empirical Bayes (BEB) posterior probability < 0.95 (Li et al. 2021; Tan et al. 2025). Second, we used the branch model (M2: model = 2, NSsites = 0; null model M0: model = 0, NSsites = 0) to detect rapidly evolving genes (REGs) in the foreground branch. Genes with a significantly higher evolutionary rate in the foreground branches than in the background branches were classified as shared rapidly evolving genes of carnivorous Characiformes. Likelihood ratio tests (LRT) were performed on the log‐likelihood (lnl) values of each model pair, and *p‐values* were derived via Chi‐square tests with a significance threshold set at *p* < 0.05.

### 2.5 Amino Acid Site Substitution Analyses

To explore whether similar genetic mechanisms underpin carnivorous adaptations in Characiformes and other fish orders, we selected *Chanodichthys erythropterus* (carnivore) and *Ctenopharyngodon idella* (herbivore) from Cypriniformes, alongside several Characiformes, for comparative genomic analysis. Using homologous coding sequences and protein sequence databases of these species, we identified amino acid substitution genes specific to carnivorous fish. Two groups were established: the IN_list included typical predatory carnivorous fish species ( *Salminus brasiliensis* , *Acestrorhynchus altus* , *Hoplias malabaricus* , *Chanodichthys erythropterus* ); the ON_list included herbivorous species ( *Colossoma macropomum* , *Megaleporinus macrocephalus*, *Distichodus sexfasciatus* , *Piaractus mesopotamicus* , *Ctenopharyngodon idella* ). To reduce false positives, genes in ON_list that share identical substitution sites with those in IN_list were filtered out. The remaining genes exhibiting identical amino acid site substitutions among carnivorous fish were retained. Substitutions at these sites were subsequently analyzed via the Convergence at Conservative Sites (CCs) method (Lyu et al. 2025; Yuan et al. 2021). The online tool SMART (Simple Modular Architecture Research Tool, https://smart.embl.de) was used to evaluate sequences with parallel amino acid substitutions, determining whether changes at the corresponding sites occurred within conserved domains. The selected amino acid substitution genes were retained, and AlphaFold (Abramson et al. 2024) (https://alphafoldserver.com) was utilized for protein 3D structure prediction. Finally, PyMOL v3.11 was employed for protein structure alignment to identify altered sites, and protein 3D structure diagrams were generated (Yang et al. 2021; Guo et al. 2021).

### 2.6 GO and KEGG Enrichment Analyses

To identify biological processes and functional pathways associated with expanded gene families, PSGs, and REGs, we conducted Gene Ontology (GO) term and Kyoto Encyclopedia of Genes and Genomes (KEGG) pathway enrichment analyses. We used InterProScan v5.16–93 (Jones et al. 2014) via the InterPro database to annotate protein sequences. Subsequently, KEGG pathway annotation was performed for these sequences. Statistical significance was assessed using the Chi‐square test, with the significance threshold set at *p* < 0.05.

## 3 Results

### 3.1 Genome Annotation

We estimated the repetitive sequence content in the annotated genomes of the five species: *A. altus* (38.97%), *D. sexfasciatus* (37.22%), *H. odoe* (32.64%), *P. mesopotamicus* (53.75%), and *P. magdalenae* (39.39%). These repetitive annotations results are generally consistent with those of previous studies. Additionally, approximately 30,000 protein‐coding genes were annotated in the five genomes: *A. altus* (29,169), *D. sexfasciatus* (33,836), *H. odoe* (28,519), *P. mesopotamicus* (32,340), and *P. magdalenae* (41,434). These gene counts are comparable to those of other closely related teleosts. BUSCO analysis of the annotation results revealed that approximately 80% of genes in the actinopterygii_odb10 gene set were identified as complete in all five species. These results confirm that our genome annotations are of sufficient quality for subsequent analyses (Table 1).

### 3.2 Orthologous Gene Identification and Phylogenetic Tree Construction

**FIGURE 1:**
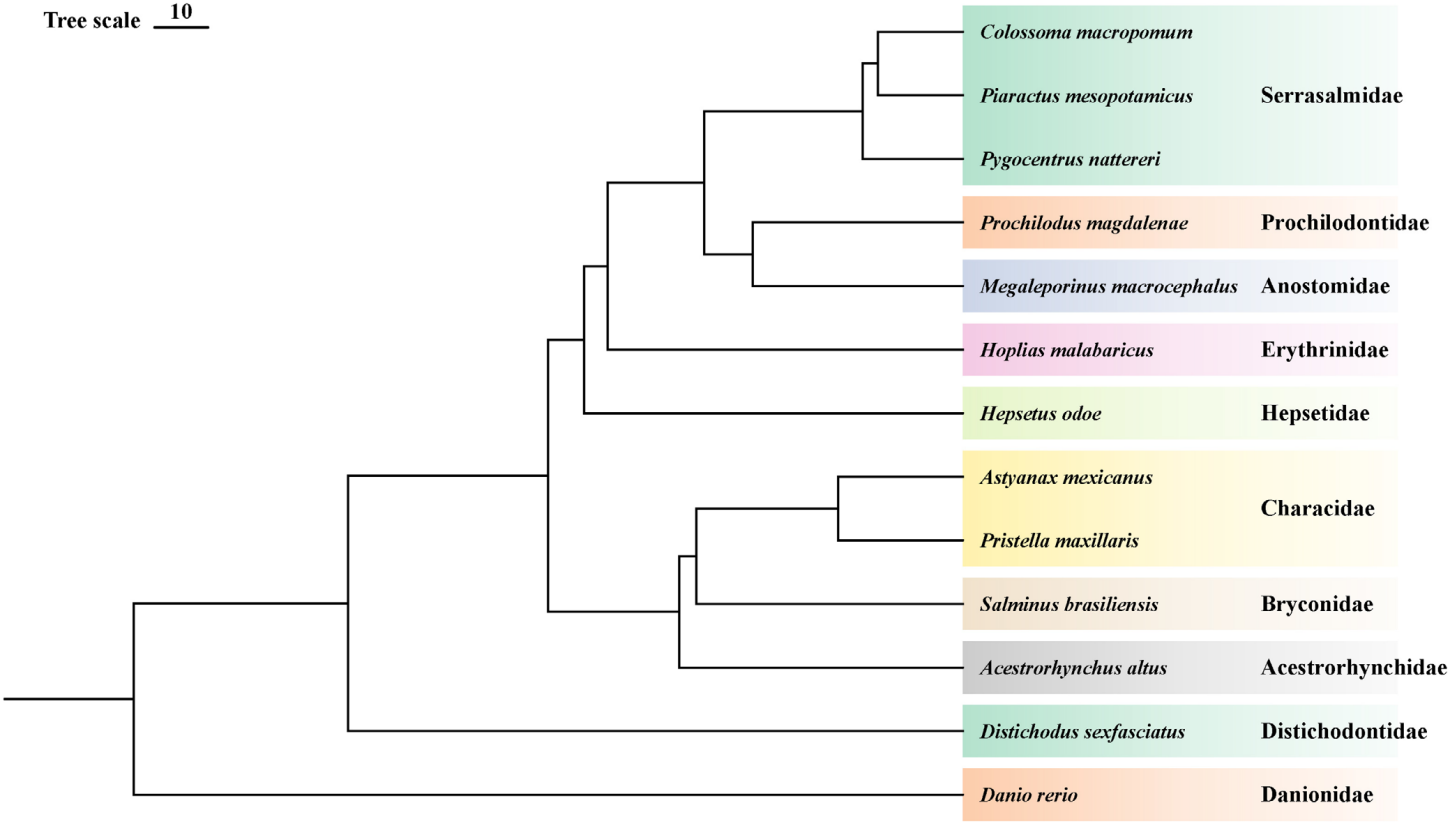
Phylogenetic tree of Characiformes species constructed based on single‐copy orthologous genes. The right side of the phylogenetic tree denotes the subfamily of each species.

**FIGURE 2:**
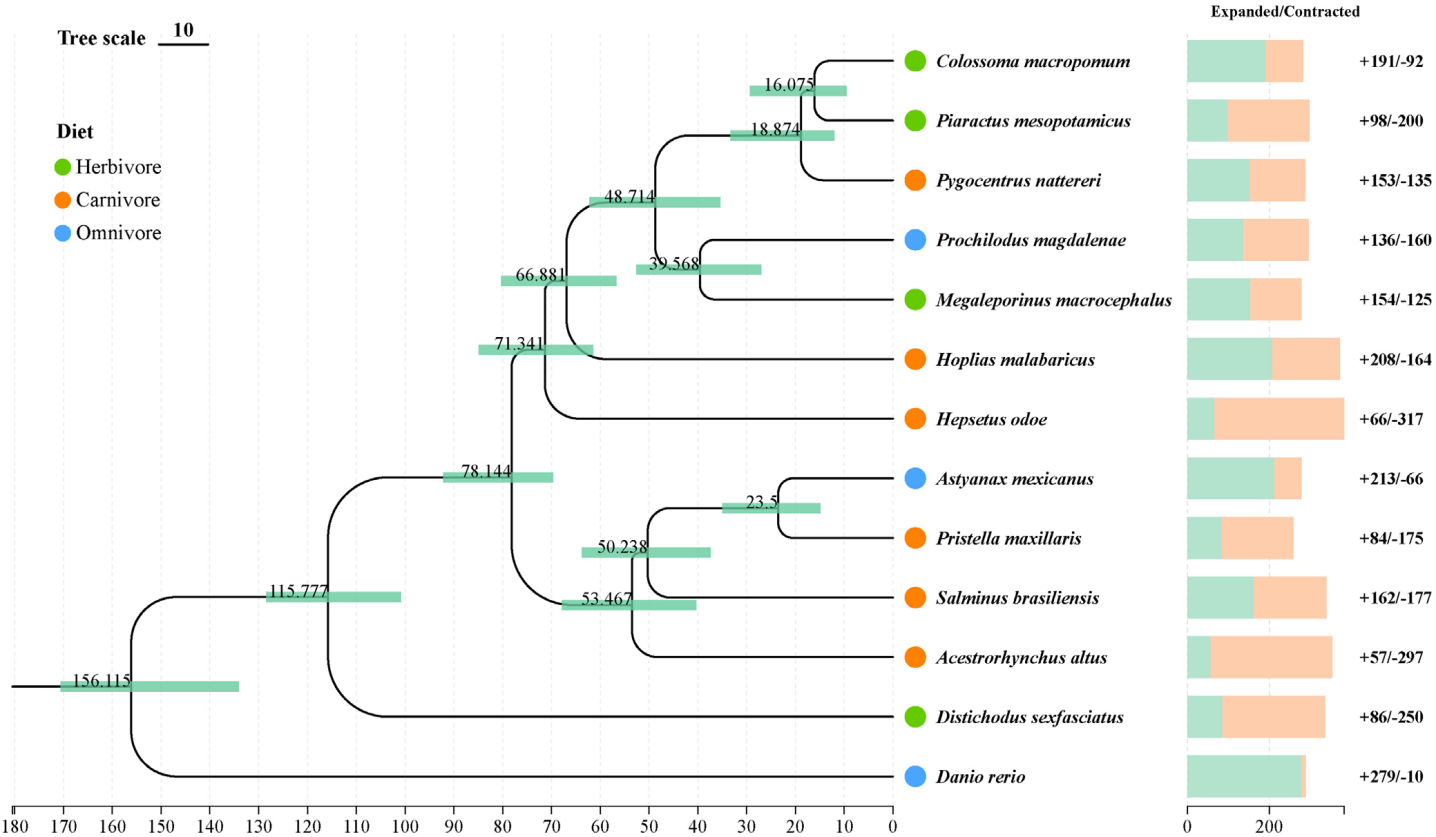
A phylogenetic tree depicting feeding habits of the studied species and gene family expansion and contraction. Circle colors denote feeding habits: Carnivory (orange), for herbivory (green), and omnivory (blue). The orange bars indicate the number of gene families that expanded during the evolution of the species and the blue bars indicate the number of gene families that contracted. The numbers above represent the species divergence time, with the unit being million years ago (Mya).

Using OrthoFinder, a total of 26,006 orthogroups were identified among 13 fish genomes. Of these, 8776 orthogroups were shared across all species, of which 3582 consisted entirely of single‐copy genes. We constructed a genome‐wide phylogenetic tree using these single‐copy genes. These 12 species cover 9 subfamilies of the order Characiformes (Figure 1). Except for Distichodontidae, which belongs to Citharinoidei, all the others are classified under Characoidei. Previous genome‐based studies involving five subfamilies within the Characoidei yielded phylogenetic relationships consistent with our findings (Yang et al. 2023). We estimated the divergence time between the two suborders Characoidei and Citharinoidei to be approximately 115.8 million years ago (Mya), which is largely consistent with two previous estimates: 129.3 Mya from earlier studies and 107.9 Mya from TimeTree. In addition, the feeding habits of these Characiformes species do not cluster based on their phylogenetic relationships (Figure 2).

### 3.3 Gene Family Identification

We counted the gene family expansions and contractions across Characiformes species (Figure 2). Among predatory species, significant expansions were identified in 57, 66, 208, 153, and 162 gene families in *A. altus* , *H. odoe* , *H. malabaricus* , *P. nattereri* , and *S. brasiliensis* , respectively. Additionally, significant contractions occurred in 297, 317, 164, 135, and 177 gene families in the same species order. Gene Ontology (GO) enrichment analysis of expanded gene families in the five carnivorous Characiformes species revealed 10 GO terms common to all five. These mainly included myosin complex (GO:0016459), heme binding (GO:0020037), cytoskeletal motor activity (GO:0003774), G protein‐coupled receptor activity (GO:0004930), and so on. Additionally, GO terms common to four of the species mainly included lipid transporter activity (GO:0005319), hemoglobin complex (GO:0005833), immune response (GO:0006955), and chitin‐related functions (chitin binding (GO:0008061) and chitin synthase activity (GO:0003950)), and so on (Table S2).

### 3.4 Positive Selection Analyses

**FIGURE 3:**
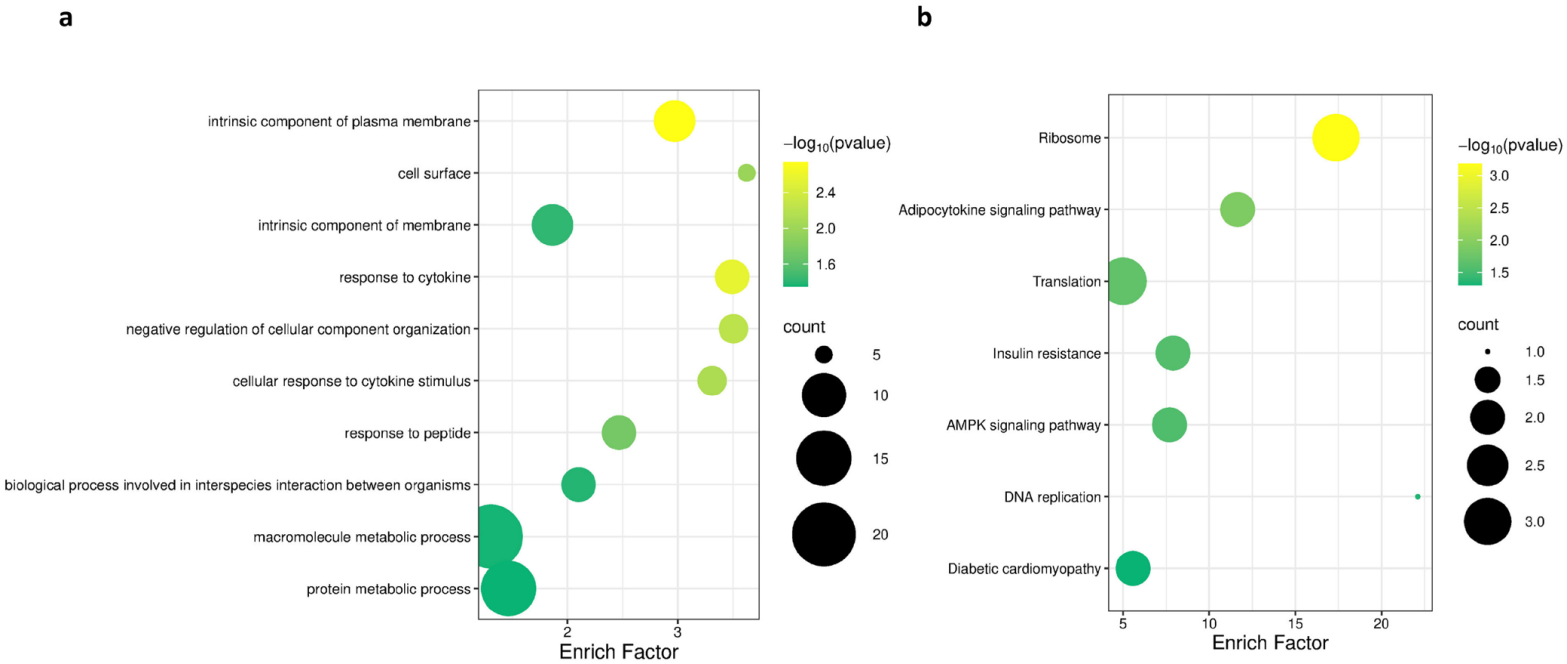
Enrichment plots of PSGs: (a) GO enrichment plot for PSGs; (b) KEGG enrichment plot for PSGs.

After screening and filtering, we finally identified 29 orthologous genes under positive selection among the five carnivorous Characiformes species (Table S3). GO enrichment analysis of these PSGs revealed that they were significantly enriched in terms including protein metabolic process (GO:0019538), response to peptide (GO:1901652), response to cytokine (GO:0034097), macromolecule metabolic process (GO:0043170), and so on. KEGG enrichment analysis further revealed that these PSGs were mainly enriched in pathways including the adipocytokine signaling pathway (ko04920), ribosome (ko03010), and AMPK signaling pathway (ko04152), and so on (Table S4 and Figure 3). Additionally, several of these positively selected genes are involved in lipid and protein metabolism and transport, as well as mitochondrial energy metabolism, collectively suggesting they play a key role in supporting the specialized high‐fat and high‐protein diet of carnivorous Characiformes.

### 3.5 Rapidly Evolving Genes Analyses

**FIGURE 4:**
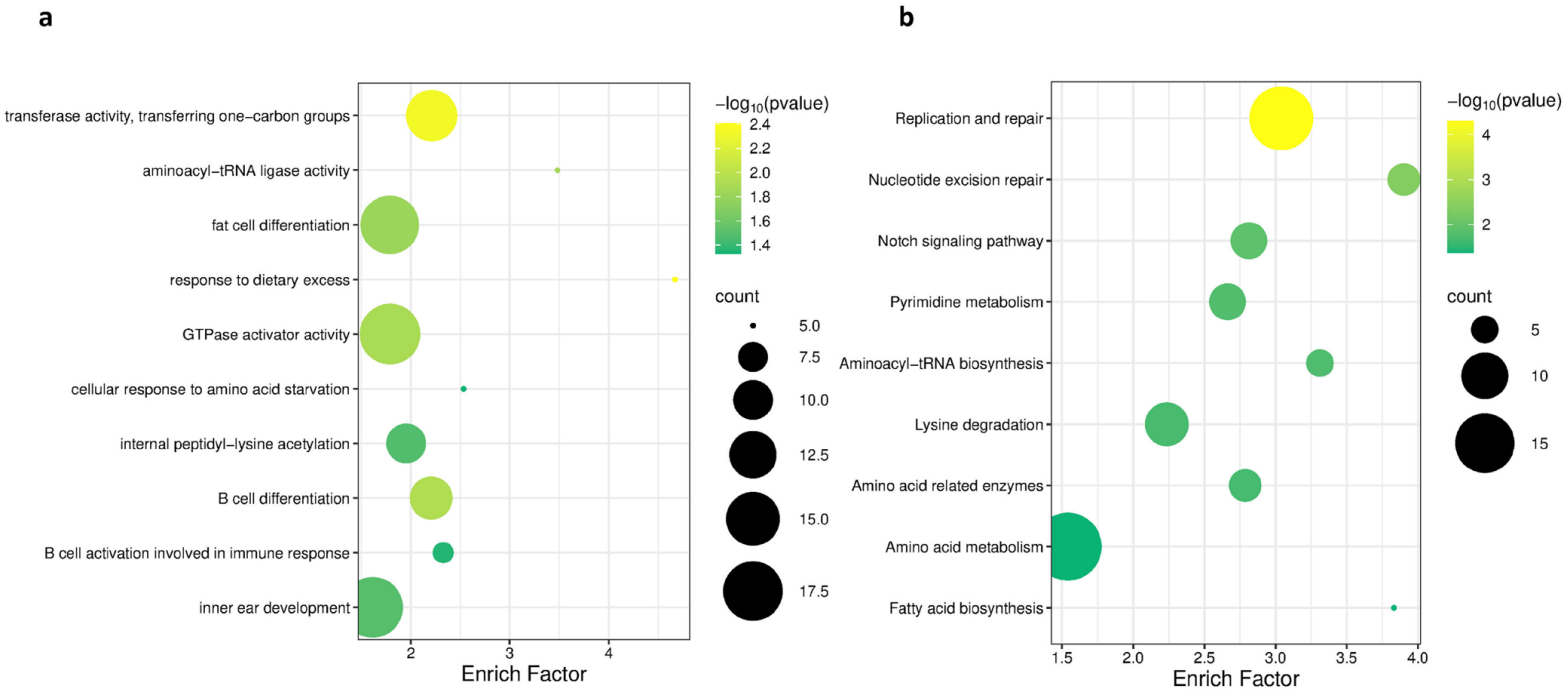
Enrichment plots of REGs: (a) GO enrichment plot for REGs; (b) KEGG enrichment plot for REGs.

Using the branch model implemented in PAML, we identified 695 REGs in ferocious carnivorous Characiformes (Table S5). GO enrichment analysis revealed that these REGs are mainly enriched in terms including nitrogen compound metabolism (cellular nitrogen compound biosynthetic process (GO:0044271), nitrogen compound metabolic process (GO:0006807)), energy metabolism regulation (nucleoside‐triphosphatase regulator activity (GO:0060589), GTPase regulator activity (GO:0030695)), fat cell differentiation (GO:0045444), cellular response to amino acid starvation (GO:0034198), and response to dietary excess (GO:0002021). This is consistent with the intermittent feeding patterns characteristic of carnivorous fish. KEGG enrichment analysis revealed that these REGs are mainly enriched in pathways including amino acid related enzymes (ko01007), amino acid metabolism (ko09105), fatty acid biosynthesis (ko00061), and lysine degradation (ko00310), and so on (Table S6 and Figure 4). Notably, eight of these REGs overlapped with the previously identified PSGs (Table 2: *avpr2aa*, *calua*, *cd79a*, *erp44*, *maf1b*, *ppargc1a*, *rtn4rl2a*, and *tsnax*).

**TABLE 2:** Overlapping genes between PSGs and REGs.

Gene	lnL(one‐ratio model)	lnL(two‐ratio model)	ω1	ω2	*p*
*avpr2aa*	−8052.433931	−8050.227062	0.14947	0.19676	0.03565064
*calua*	−3834.889689	−3830.856495	0.02672	0.05400	0.004509392
*cd79a*	−5616.687395	−5612.184921	0.45934	0.72436	0.002692497
*erp44*	−5079.939348	−5077.009609	0.06725	0.10572	0.015493388
*maf1b*	−2616.210118	−2613.102067	0.02824	0.05596	0.012659353
*ppargc1a*	−8961.544009	−8957.481434	0.24301	0.34983	0.004365537
*rtn4rl2a*	−5723.916209	−5720.171689	0.05028	0.08127	0.006207564
*tsnax*	−4643.99952	−4638.188861	0.09101	0.16458	0.000652002

### 3.6 Amino Acid Site Substitution Analyses

**FIGURE 5:**
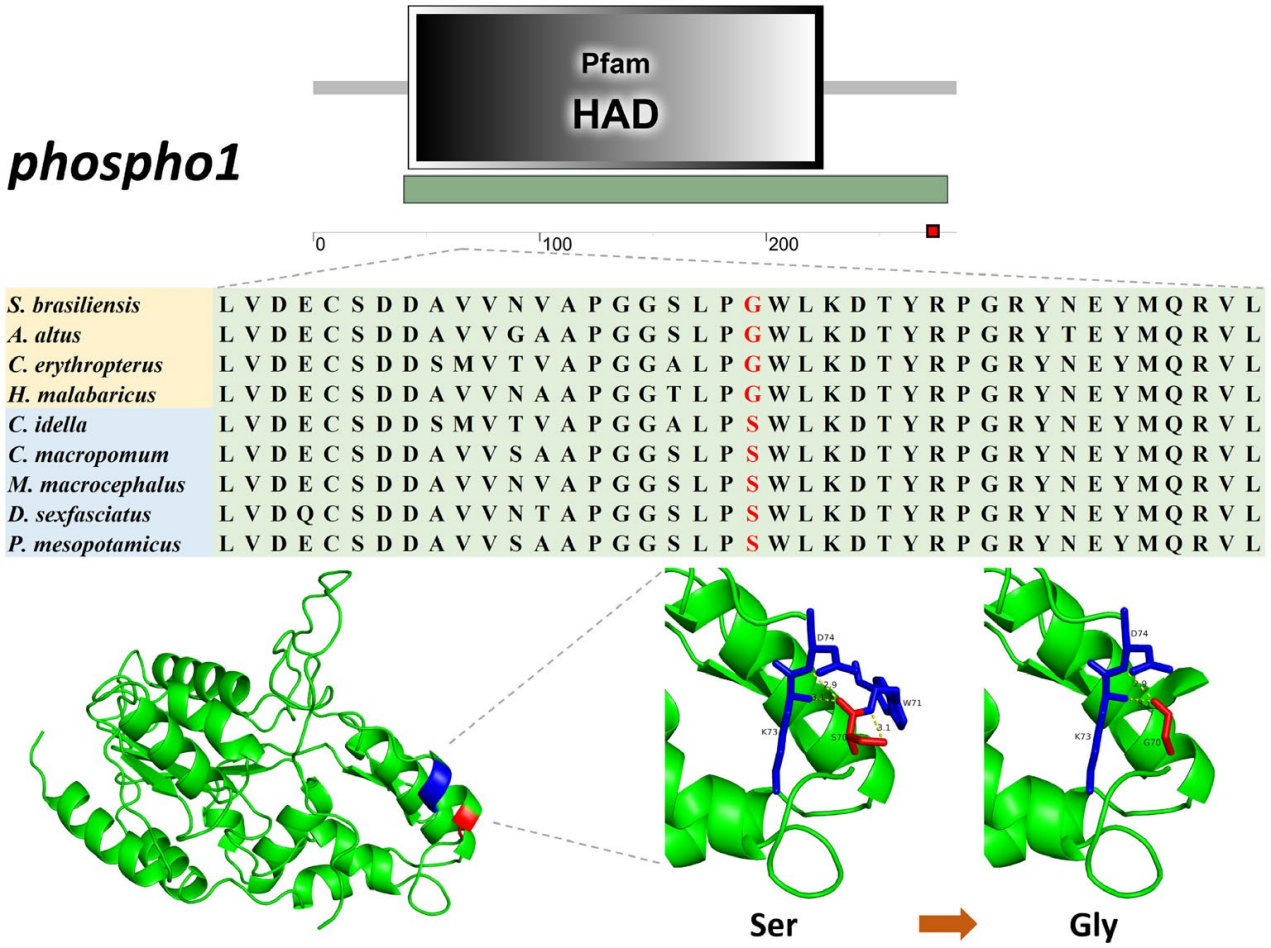
Amino acid substitution site in the *phospho1* gene and 3D structure diagram of its encoded protein.

**FIGURE 6:**
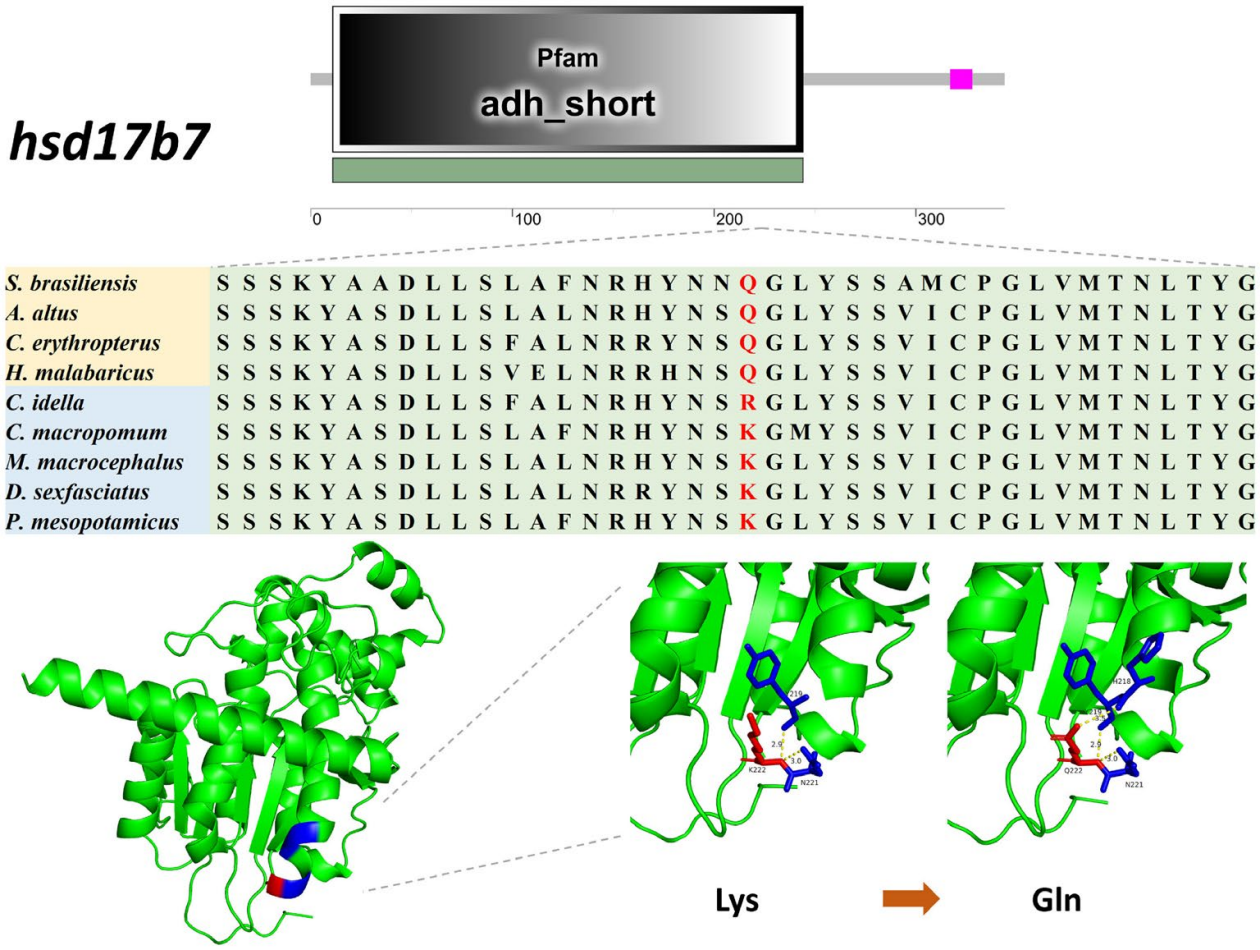
Amino acid substitution site in the *hsd17b7* gene and 3D structure diagram of its encoded protein.

After filtering analysis, we removed genes in the ON_list that shared the same substitution sites as genes in the IN_list. We ultimately identified 153 genes in the IN_list harboring identical amino acid substitutions. Domain analysis of their encoded proteins revealed that 52 of these genes had substitution sites within conserved domains (Table S7). Subsequently, we searched and reviewed the functions of these genes, and ultimately identified three genes (*enpep*, *hsd17b7*, *phospho1*) whose functions are potentially related to carnivorous adaptation and supported by both literature and experimental evidence. After 3D protein structure prediction, we identified two genes—*phospho1* and *hsd17b7*—wherein amino acid substitutions caused significant structural changes. The two amino acid substitution sites in the *enpep* gene did not cause significant changes in its protein structure. *Phospho1* harbors a single amino acid substitution at position 71, with the hydrophilic Ser (S) substituted by the hydrophobic Gly (G). *Hsd17b7* has a substitution at position 222, where the basic residues Lys (K) and Arg (R) are both replaced by the neutral Gln (Q). The 3D structural diagrams of the proteins encoded by these two genes show that these substitutions alter the intramolecular interactions among amino acids within the proteins, leading to structural changes (Figure 5 and Figure 6). These structural changes are inferred to potentially affect their biological functions.

## 4 Discussion

Teleost fishes represent the most evolutionarily successful group among aquatic animals, exhibiting extensive diversity in morphology, feeding habits, metabolism, and other traits to adapt to complex aquatic environments (Gui et al. 2022). Regarding feeding habits, previous studies have examined adaptations in feeding behavior and metabolic characteristics between carnivorous and herbivorous fish through organ features and morphology, respectively (Hofer and Schiemer 1981; He et al. 2013). However, research into the molecular mechanisms underlying these dietary adaptations remains limited. Moreover, as a significant group of Teleost fishes, Characiformes comprise numerous species with diverse feeding habits, making them an excellent model for studying species evolution and trait diversity that deserves increased scientific attention. In this study, we employed the comparative genomics approach to analyze the molecular mechanisms of carnivorous fishes in Characiformes, providing fundamental insights into the evolution of carnivorous fishes and their adaptive characteristics in terms of feeding habits and metabolism.

Whether the order Characiformes is monophyletic or paraphyletic has long been one of the most contentious issues in fish systematics. In earlier studies, most ichthyologists hypothesized that Characiformes are monophyletic based on striking similarities in anatomy, morphology, and ecology (Chakrabarty et al. 2017). However, recent studies using ultra‐conserved elements (UCEs) and genomic data have increasingly supported the classification of Characiformes as a paraphyletic group, consisting of the suborders Characoidei and Citharinoidei (Yang et al. 2023). In our study, phylogenetic relationships among subfamilies of Characiformes are consistent with those reported in previous studies. Notably, a recent study used whole‐genome alignment data to reconstruct the phylogenetic relationships of teleost fishes, encompassing all ordinal lineages. This phylogeny also supports the classification of Characiformes as a paraphyletic group, with the divergence time between its two suborders estimated at ~79.02 Mya (Song et al. 2025). This date is earlier than our inferred divergence time, likely because only one Citharinoidei genome was included in our analysis.

In our study, the feeding habits of these Characiformes species did not cluster according to their phylogenetic relationships. Diet is arguably one of the most potent selective pressures acting on all species on Earth. In particular, carnivory has evolved independently multiple times across numerous mammalian lineages. Indeed, many highly specialized carnivorous species have closely related counterparts with far more diverse feeding habits: for example, within the Ursidae, the polar bear, the omnivorous grizzly, and the herbivorous panda. This pattern highlights that evolutionary lability in feeding habit is widespread across animal taxa (Kim et al. 2016). In a study of African crater lake cichlid fish, two ecomorphs of *Astatotilapia* were described, which exhibit extremely low genome‐wide sequence divergence but pronounced differences in morphology and feeding habit. The study proposed that these *Astatotilapia* populations first colonized shallow lake habitats approximately 10,000 years ago, and subsequently colonized benthic habitats around 1000 years ago. Reversible and heritable methylation differences in key functional genes, including those involved in hemoglobin synthesis, erythropoiesis, and sterol metabolism, likely mediated their adaptation to the hypoxic conditions and zooplankton‐rich resource environment of the lake's deep water habitats (Vernaz et al. 2022). Meanwhile, in a study on the feeding habits of Citharinoidei, ancestral state reconstruction of feeding preferences revealed that planktivore, omnivore, and invertivore emerged early in the evolutionary history of Citharinoidei. Subsequently, herbivore, piscivore, and pterygophagy evolved independently from invertivore ancestors. These results indicate that such dietary shifts can occur over relatively short evolutionary time spans (Lavoué et al. 2017). Collectively, these studies demonstrate that dietary differentiation is a major driver of fish diversification. We speculate that during the evolutionary process of Characiformes, shifts in feeding habit have primarily occurred to avoid interspecific competition and adapt to the distinct ecological niches occupied by each lineage. This has resulted in divergent feeding habits among closely related species. We anticipate that with the availability of more Characiformes genomes and their inclusion in future analyses, the divergence times of its respective subfamilies will be determined with higher precision, and the evolutionary processes of more refined dietary classifications can be comprehensively explored.

Feeding habits are closely associated with metabolic adaptations (Cheng et al. 2023). Carnivorous animals are known to consume a high‐protein, high‐fat, and low‐carbohydrate diet. By contrast, research has shown that herbivorous species as adults exhibit significantly lower specific protease activity, reduced protein metabolism, and lower dietary protein requirements compared to their carnivorous counterparts (Hofer and Schiemer 1981). In our study, enrichment analyses of PSGs and REGs were enriched in multiple pathways associated with protein metabolism. Comparative genomic analysis of *Leiocassis longirostris* Günther, a typical carnivorous fish, showed that its expanded gene families, PSGs, and REGs were similarly enriched in pathways involved in amino acid and protein synthesis and metabolism (Liu et al. 2024). Combined with our results, genes related to amino acid metabolism such as protein digestion and absorption represent important molecular characteristics of dietary adaptation in carnivorous fish. We identified the *rhbg* gene among the PSGs, which facilitates ammonia excretion through fish gills. In gene knockout experiments targeting the zebrafish Rh protein family, zebrafish deficient in *rhbg* showed significant inhibition of ammonia transport (Braun et al. 2009). A study on Yellow River Carp showed that when dietary protein levels increased from 25% to 31%, the relative expression of *rhag*, *rhbg*, and *rhcg1* was significantly upregulated. This indicates that elevated dietary protein levels promote ammonia metabolism in Yellow River Carp (Wang et al. 2023). We hypothesize that *rhbg* may be involved in excreting ammonia metabolites resulting from high‐protein diets in carnivorous fish.

Similarly, lipid transport and metabolism‐related pathways were enriched in expanded gene families and REGs. Gene family expansion is often accompanied by an increase in gene copy number. In a study on copy number variations of dietary‐related genes in mammals, the results confirmed that omnivorous mammals had higher copy numbers of the amylase gene than herbivores and carnivores, whereas herbivores exhibited higher copy numbers of several olfactory receptor genes and genes encoding enzymes involved in xenobiotic detoxification (Wilhoit et al. 2025). Our functional enrichment analysis revealed that the expanded gene families in the four carnivorous fish species were enriched for lipid transporter activity (GO:0005319). An obligate carnivorous diet implies that carnivorous fish must process a greater fat load compared with fish of other feeding habits. We speculate that these fish may improve the efficiency of fat utilization by increasing the copy number of lipid transporter genes. Among the PSGs, we identified *cd36*—a gene encoding a long‐chain fatty acid‐binding protein. CD36 is expressed in numerous tissues and organs, facilitating cellular uptake of long‐chain fatty acids. Studies have shown that mice fed a 40% fat diet exhibited 3.5‐fold higher CD36 expression in cardiac tissue than those on a 9% fat diet (Greenwalt et al. 1995). Furthermore, CD36 protein levels were significantly elevated in the livers of diet‐induced obese mice, suggesting its association with enhanced hepatic triglyceride storage and secretion (Koonen et al. 2007). We speculate that under the selective pressure of a high‐fat diet, the *cd36* gene in carnivorous fish can enhance protein expression, thereby improving lipid absorption and conversion. Cholesterol 7α‐hydroxylase (*cyp7a1*, a REG) is the rate‐limiting enzyme in the bile acid biosynthesis pathway, catalyzing the conversion of cholesterol to bile acids in the liver. Research has found that transgenic mice overexpressing *cyp7a1* in the liver are resistant to obesity, fatty liver disease, and insulin resistance induced by a high‐fat diet. These findings indicate that *cyp7a1* plays a crucial role in maintaining lipid and energy homeostasis (Li et al. 2010). In addition, among the two genes with amino acid site substitutions identified in our study, the bone mineralization phosphatase, also known as phosphatase, orphan 1 (PHOSPHO1), has been demonstrated to play a role in metabolic regulation in mice and humans, and is regarded as a potential therapeutic target for obesity and diabetes (Dillon et al. 2019; Chambers et al. 2015; Wu et al. 2018). *Phospho1* knockout led to decreased blood glucose levels, increased insulin sensitivity and enhanced glucose tolerance in juvenile, adult and aged mice, indicating an improvement in basal glucose homeostasis. Following high‐fat diet feeding, *Phospho1* deficiency still exerted significant protective effects against diet‐induced obesity, diabetes and non‐alcoholic fatty liver disease (NAFLD) (Suchacki et al. 2020; Oldknow and Suchacki 2016). Another study found that *phospho1*‐knockout mice exhibited enhanced cold tolerance and higher expression levels of thermogenic genes in brown adipose tissue (BAT), suggesting that *phospho1* plays a role in thermogenesis and energy metabolism in BAT. Consistently, these mice were also protected against high‐fat diet‐induced obesity and insulin resistance (Jiang et al. 2020). The 17β‐hydroxysteroid dehydrogenases (17β‐HSDs) constitute a family of 15 members. They play key roles in sex hormone metabolism by catalyzing steps in steroid biosynthesis, and some members may also function in cholesterol and fatty acid metabolism (Wilson et al. 2025). *Hsd17b7* encodes a key enzyme in cholesterol biosynthesis. In mice, high‐fat diet‐induced obesity upregulates the gene expression of *hsd17b7* and *hsd17b11* in the liver. To clarify the metabolic role of this enzyme in vivo, another study generated *hsd17b7*‐knockout mice lacking enzymatic activity. The results showed that deletion of *hsd17b7* led to impaired *de novo* cholesterol biosynthesis in mouse embryos (Jokela et al. 2010). During the dietary adaptation of *Micropterus salmoides* , *hsd17b7* and other steroid biosynthesis‐related genes were upregulated in individuals that readily accepted artificial pellet feed. This indicates that *hsd17b7* plays a significant role in the early stages of dietary domestication in *M. salmoides* , enhancing lipid utilization efficiency of artificial pellet feed (Du et al. 2023). Compared to herbivorous fishes, these two genes in carnivorous fishes harbor amino acid substitutions at specific sites. In the encoded proteins, an additional hydrogen bond has been formed, establishing new interactions with neighboring amino acids. This is coupled with significant alterations in protein structure, which may modify their original biological functions. These genes likely represent key molecular mechanisms allowing carnivorous fish to more efficiently absorb and transport lipids, and thereby overcome the adverse effects of high‐fat diets and maintain metabolic balance.

Notably, we identified 8 genes that are both PSGs and REGs. In *L. longirostris* , the *tas1r3* and *trypsin* genes were simultaneously identified through both PSGs and REGs, providing insights into its carnivorous feeding preference and corresponding metabolic strategies (Liu et al. 2024). Peroxisome proliferator‐activated receptor‐gamma coactivator 1α, encoded by *ppargc1a*, acts as a transcriptional coactivator that regulates energy homeostasis. Furthermore, *ppargc1a* is involved in nitric oxide‐induced mitochondrial biogenesis, while promoting a shift in muscle metabolism toward oxidative phosphorylation, thereby enhancing muscle tissue endurance (Mazur et al. 2020). We speculate that this may be a key factor underlying the explosive energy supply and powerful muscles exhibited by carnivorous fish during hunting. Previous studies have reported that hepatic insulin sensitivity is enhanced in *ppargc1a*‐deficient mice, while a separate study demonstrated that hepatic *ppargc1a* expression is elevated in mice fed a high‐fat diet (Koo et al. 2004; Park et al. 2014). These in vivo expression validation experiments in mouse models collectively support that *ppargc1a* exerts a key regulatory role in lipid metabolism (Zhang et al. 2020). Collectively, these findings lead us to speculate that *ppargc1a* may represent an important molecular foundation for the adaptive evolution of carnivory in these Characiformes species. Chitin is a naturally occurring linear polysaccharide with a chemical structure analogous to cellulose. It is widely distributed in the exoskeletons of invertebrates (e.g., the exoskeletons of shrimp, crabs, and insects). The pathways enriched in expanded gene families, including chitin binding (GO:0008061) and chitin synthase activity (GO:0003950), may be associated with carnivorous fish's predation on insects and crustaceans.

Finally, due to the scarcity of publicly available, high‐quality genome data for Characiformes fishes, our analysis could only roughly classify feeding habits into carnivorous, herbivorous, and omnivorous types, and was unable to incorporate more detailed categories of other specialized feeding habits such as pterygophagy. Additionally, our study only includes one species from the suborder Citharinoidei. These limitations in genome data may affect the comprehensiveness of our conclusions regarding the evolution of feeding habits in the phylogeny of Characiformes. We believe that in the future, with the increasing availability and completion of more high‐quality Characiformes genomes, the analysis of feeding habits in this group will become more refined and comprehensive, facilitating better research on this diverse taxon.

## 5 Conclusions

In this study, we reannotated the genomes of five Characiformes species and performed a genome‐wide analysis of feeding habit‐related adaptations. Comparative genomic analysis revealed that specialized carnivory in Characiformes is primarily linked to pathways related to amino acid metabolism, lipid absorption and transport, and energy metabolism. We also identified candidate genes including *rhbg*,*cd36*, *cyp7a1*, *phospho1*, *hsd17b7*, and *ppargc1a*, providing a foundation for future functional validation experiments. These findings elucidate, from pathways to individual genes, how carnivorous Characiformes efficiently absorb and utilize high‐protein, high‐fat diets while counteracting their adverse effects. They offer direct evidence for the molecular mechanisms underlying the adaptive evolution of carnivory in Characiformes.

## Author Contributions

**Shihu Zhao:** conceptualization (lead), data curation (lead), formal analysis (equal), investigation (lead), methodology (lead), software (equal), visualization (lead), writing – original draft (lead), writing – review and editing (equal). **Xuesong Mei:** data curation (equal), formal analysis (equal), methodology (equal), software (equal), writing – review and editing (equal). **Zhihao Zhang:** data curation (equal), formal analysis (equal), methodology (equal), software (equal), writing – review and editing (equal). **Tian Xia:** formal analysis (equal), software (equal), writing – review and editing (equal). **Shengyang Zhou:** methodology (equal), software (equal). **Yuehuan Dong:** data curation (equal), visualization (equal). **Shangbin He:** data curation (equal), visualization (equal). **Zhicheng Yao:** supervision (equal), visualization (equal). **Yuchun Li:** supervision (equal), visualization (equal). **Xiufeng Yang:** project administration (equal), supervision (equal), writing – review and editing (equal). **Honghai Zhang:** funding acquisition (lead), project administration (lead), writing – review and editing (equal).

## Funding

This research was supported by the National Natural Science Foundation of China (32270444, 32470448), and the Natural Science Foundation of Shandong Province (ZR2023ZD47).

## Ethics Statement

The authors have nothing to report.

## Consent

The authors have nothing to report.

## Conflicts of Interest

The authors declare no conflicts of interest.

## Supporting information


**Table S1:** Information on publicly available genomes of Characiformes.

**Table S2:** GO enrichment results of expanded gene families in five carnivorous Characiformes species species.

**Table S3:** Positively selected genes in five carnivorous Characiformes species.

**Table S4:** GO and KEGG enrichment results of positively selected genes in five carnivorous Characiformes species.

**Table S5:** Rapidly evolving genes in five carnivorous Characiformes species.

**Table S6:** GO and KEGG enrichment results of rapidly evolving genes in five carnivorous Characiformes species.

**Table S7:** Genes with amino acid substitutions (153 genes in total), of which 52 genes had mutations located in conserved domains.

## Data Availability

All genome sequences used in this study are available on the NCBI database (https://www.ncbi.nlm.nih.gov) under the accession numbers reported in Table 1.
